# Editorial: Incisional Hernia Prevention

**DOI:** 10.3389/jaws.2023.11495

**Published:** 2023-05-05

**Authors:** Manuel López-Cano

**Affiliations:** General Surgery/Abdominal Wall Surgery, Vall d'Hebron University Hospital, Barcelona, Spain

**Keywords:** incisional hernia, laparotomy, prophylaxis, parastomal, trocar

Incisional hernia (IH) is a health problem of the first order, with a significant impact on the lives of patients who suffer from it and with high economic and social costs at all levels. Therefore, it is not surprising that in the last decade many efforts have been dedicated to knowing more and better all the aspects related to its prevention. Most of these aspects are covered with the articles published in this Special Issue about the prevention of IH and they can be divided into general and specific aspects.

Among the general aspects: the definition of high-risk patients who may benefit from the prevention of IH (Pereira-Rodríguez et al.); innovations in prostheses for the prevention of IH (Harris); analysis of the best non-mesh closure technique for an elective midline laparotomy (Fortelny) or what is the degree of implementation of prosthetic meshes in the prevention of IH (Durbin et al.). Other more specific aspects can be: IH prevention at trocar sites when using minimally invasive surgical techniques (de Beaux and East); anatomical location in the abdominal wall of a laparotomy and its influence on the opening and/or closure of the abdominal wall (Medina Pedrique et al.) or how can IH be prevented in the context of oncological diseases that require cytoreductive surgery and Hyperthermic Intraperitoneal Chemotherapy (HIPEC) (Wenzelberg et al.).

The increase in knowledge about the prevention of IH and how to apply it at general and specific levels may lead to a greater increase in the cost-effectiveness of abdominal surgery, a reduction in morbidity, and better health-related quality-of life of our patients. We are aware that even today the late complications of surgeries that require opening and closing of the abdominal wall (i.e., IH) tend to be relegated to the background when the safety of a specific intraabdominal surgical intervention is evaluated. However, these complications (i.e., IH) play a decisive role in the quality of life of the patient and in the costs of the process and we must prevent them as much as possible.

Although this Special Issue does not cover all the aspects that can be considered in the prevention of IH, it does offer a fairly comprehensive overview. We hope it will be helpful to interested readers and help improve the prevention of IH in patients requiring laparotomy.

